# Antibacterial Properties of Silver Nanoparticles Embedded on Polyelectrolyte Hydrogels Based on α-Amino Acid Residues

**DOI:** 10.3390/gels4020042

**Published:** 2018-05-04

**Authors:** Mario Casolaro, Ilaria Casolaro, Jun Akimoto, Motoki Ueda, Masashi Ueki, Yoshihiro Ito

**Affiliations:** 1 Dipartimento di Biotecnologie, Chimica e Farmacia (Dipartimento di Eccellenza 2018–2022), Università degli Studi di Siena, via A. Moro, 2-53100 Siena, Italy; 2 ASST Valtellina e Alto Lario, Dipartimento di Salute Mentale, via Stelvio, 25-23100 Sondrio, Italy; casolaro.ilaria@gmail.com; 3 Nano Medical Engineering Laboratory, Riken, 2-1 Hirosawa, Wako, Saitama 351-0198, Japan; jun.akimoto@riken.jp (J.A.); motoki.ueda@riken.jp (M.U.); uemasa@riken.jp (M.U.); y-ito@riken.jp (Y.I.)

**Keywords:** polyelectrolyte hydrogels, silver nanoparticles, equilibrium degree of swelling, TEM, antimicrobial activities

## Abstract

Polyelectrolyte hydrogels bearing l-phenylalanine (PHE), l-valine (AVA), and l-histidine (Hist) residues were used as scaffolds for the formation of silver nanoparticles by reduction of Ag^+^ ions with NaBH_4_. The interaction with the metal ion allowed a prompt collapse of the swollen hydrogel, due to the neutralization reaction of basic groups present on the polymer. The imidazole nitrogen of the hydrogel with Hist demonstrated greater complexing capacity with the Ag^+^ ion compared to the hydrogels with carboxyl groups. The subsequent reduction to metallic silver allowed for the restoration of the hydrogel’s degree of swelling to the starting value. Transmission electron microscopy (TEM) and spectroscopic analyses showed, respectively, a uniform distribution of the 15 nm spherical silver nanoparticles embedded on the hydrogel and peak optical properties around a wavelength of 400 nm due to the surface plasmonic effect. Unlike native hydrogels, the composite hydrogels containing silver nanoparticles showed good antibacterial activity as gram+/gram− bactericides, and higher antifungal activity against *S. cerevisiae*.

## 1. Introduction

**Scheme 1:**
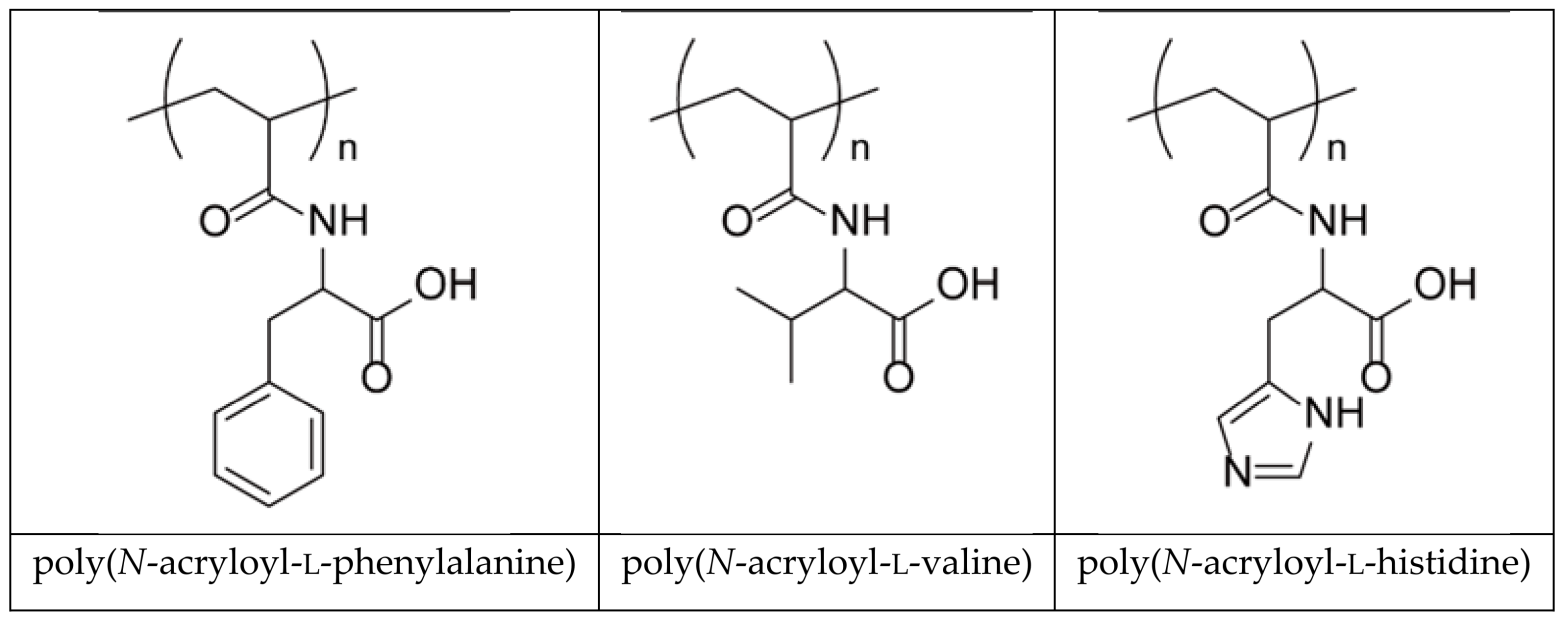
Structure of poly(*N*-acryloyl-l-phenylalanine), poly(*N*-acryloyl-l-valine), and poly(*N*-acryloyl-l-histidine).

Today it is widely accepted that polymeric materials are extremely useful in various fields of science and technology. In particular, cross-linked polymers (hydrogels) play a fundamental role for various biomedical purposes due to their compatibility with soft tissues [1,2,3]. Since hydrogels have structural and compositional similarities with respect to extracellular matrices and their broad structure for cell proliferation and survival, they have for a long time attracted considerable attention in biomedicine. Hydrogels are materials capable of retaining a large amount of water inside their porous structure and are therefore subject to deformation due to external triggers [3]; among these we can include direct stimuli (pH, temperature, ionic strength) or remote stimuli (alternating magnetic field, light). A series of polyelectrolyte hydrogels bearing α-amino acid residues (l-valine, l-phenylalanine, l-histidine), which better approximate the properties of biological tissues consisting of filamentous polyelectrolyte proteins, have been studied for many years (Scheme 1) [3,4,5].

The presence of ionic/ionizable functional groups (carboxyl, amine) allows these hydrogels to complex metal ions and/or drug molecules, also based on metals, for their better use in controlled release in different and relevant therapies [6]. The hydrogels behave as containers of drugs that, in addition to preserving the drug itself from possible degradation, control its release kinetics in the surrounding environment by virtue of the polymer–drug ionic interaction force. The studies previously reported for the release of cisplatin [7,8] and other metal-based drugs [9,10], as well as the release of anticancer drugs (doxorubicin) [11], treatment of glaucoma (pilocarpine) [12], and antidepressants (trazodone, citalopram, paroxetine, duloxetine, vortioxetine) [13,14,15], confirm that the main polymer–drug interaction is ionic in nature; the strength of this interaction influences the whole release process, with the free drug molecule, in equilibrium with the adduct, which diffuses through the hydrogel network to be finally used for therapeutic purposes. The strength of this interaction is attributed to the basicity constants (pKa) values of the ionic groups involved in the process: carboxyl group of the hydrogel and amine group of the drug. In general, and with the same hydrogel in the swollen state, a greater pKa of the cationic drug produces a more rapid and complete collapsing phenomenon.

In this paper we propose to report the behavior of the hydrogels mentioned above against the silver ion in solution and its subsequent reduction to metallic silver in the form of nanoparticles. Silver nanoparticles, used in a variety of technologies, are incorporated into numerous consumer products to benefit from their unique conductive, optical, and primarily antibacterial properties [16]. Silver nanoparticles have become increasingly popular for antimicrobial coatings, especially in biomedical devices, as the continuous release of a low level of silver ions provides protection against bacteria [17,18]. As is well known, biofouling, i.e., the accumulation of bacteria on material surfaces, is a worldwide problem that many researchers have taken into account. Various antibacterial reagents such as chitosan, quaternary ammonium salts, and metal ions are employed to enhance the antibacterial properties of membranes [19,20]. Some years ago, we developed a new type of copolymer composed of l-histidine (ampholyte) and *n*-butyl methacrylate (hydrophobic moiety) for the preparation of nonbiofouling surfaces [21]. More recently, Wang et al. proposed a straightforward mussel-inspired approach to construct chitosan–polyurethane coatings and load Ag nanoparticles to endow polyethersulfone membrane with dual antibacterial and antifouling properties [22]. Moreover, a substrate-independent Ag-nanoparticle-loaded temperature-sensitive hydrogel coating was synthesized and covalently attached onto a model substrate. The coating showed good hemo- and cytocompatibility, and the effect of temperature revealed cell detachment properties on various medical devices [23]. To date, a universal approach for fabricating low-cost antifouling surfaces is based on tannic acid and a novel reactive zwitterionic polymer [24].

The use of hydrogels, synthetic and natural, can contribute to the development of theranostic agents for more specific clinical therapies [25]. The development of microcontainers is attractive for applications in biomedicine and cell biology. Such an example of biocompatible hollow microcapsules, alginate matrix, and silver nanoparticles, where the silver alginate hydrogel microcapsules serve as microcontainers with a functional substance payload, has recently been reported by Lengert et al. [26]. As well as other synthetic hydrogels, based on acrylates, they were studied as composites and multifunctional orthopedic hydrogels such as osseoconductive and non-antibiotic based antibacterial [27]. Also, other nanocomposites based on acrylic copolymers and containing ionic units, such as sulfonic and carboxyl groups, which are capable of attracting a greater number of silver ions, have shown good performance with respect to antibacterial activity [28,29]. Moreover, the presence of ionic groups on the polymer can mitigate the stabilization of Ag nanoparticles, decreasing the surface energy; once the nanoparticles in solution have been produced, the ionic polymeric units associate with the surface of the nanoparticles to establish a double layer of charge that stabilizes the particles themselves and prevents their aggregation. It is known that the complexing ability of silver ion with the amino group is stronger than that of the carboxyl group, and this should induce a greater stabilization of the obtained nanoparticles [30]. As in the case of bovine albumin, groups carrying sulfur, oxygen, and nitrogen mitigate the high surface energy of the nanoparticles during the reduction; moreover, the hydroxyl groups on cellulose help stabilize the particles. It is to be expected that the various hydrogels used in this work can behave this way and therefore, to varying degrees, can mitigate the stabilization of the silver nanoparticles obtained.

## 2. Results and Discussion

### 2.1. Protonation and Complex Formation Thermodynamics

**Figure 1:**
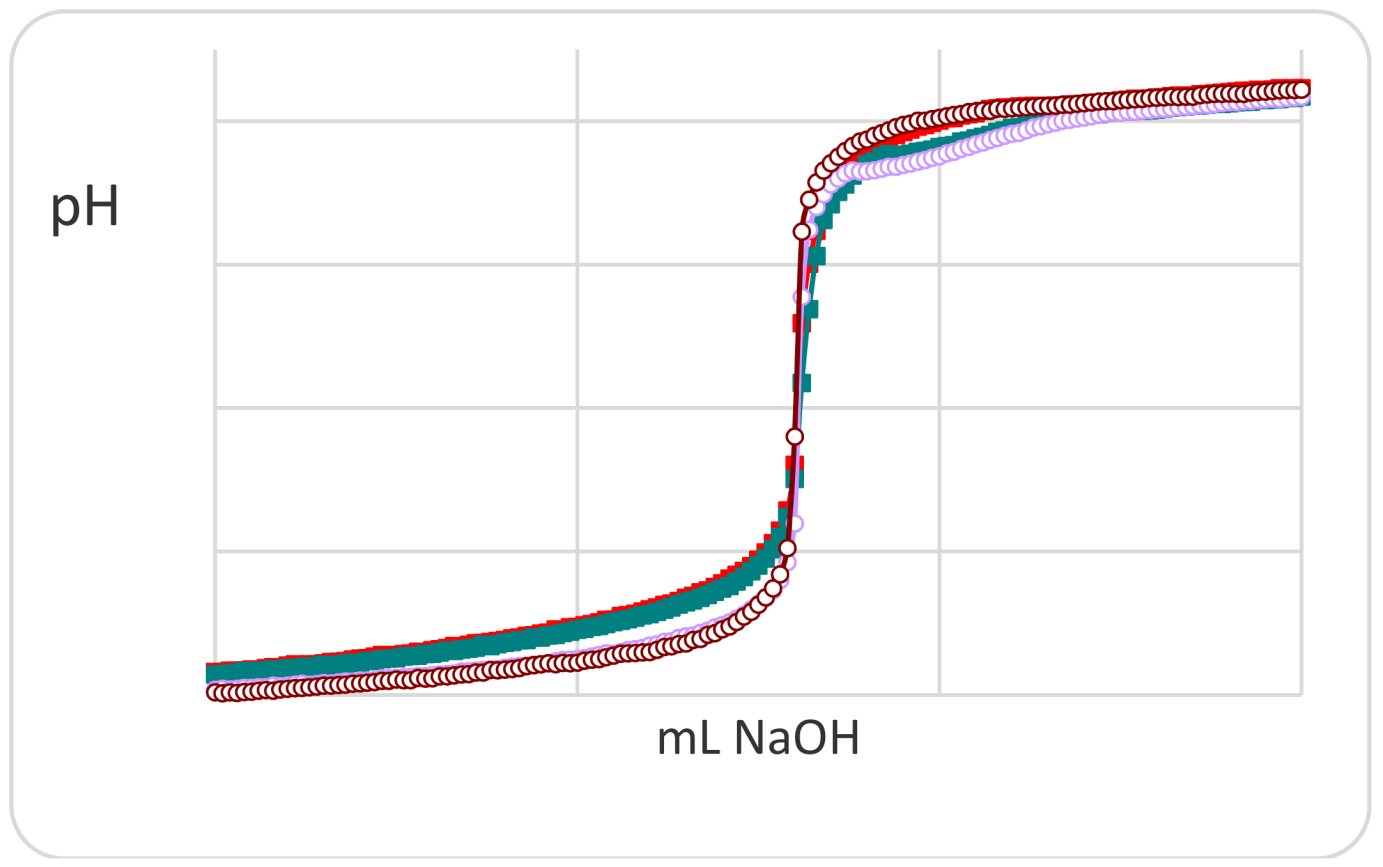
Potentiometric titration curves of monomer *N*-acryloyl-l-phenylalanine (PHE) in the absence (red filled) and in the presence (green filled) of Ag(I) ions. For comparison, the titration curves of nitric acid (brown open) and silver(I) ions (violet open) are also reported.

A preliminary potentiometric titration study for understanding the interaction strength of the different ionic groups present on the polymer was carried out mainly on the monomer precursors. The latter, being more soluble than the corresponding polymers, can give more information about the complexing ability towards silver ions in a wide pH range. As was to be expected, the *N*-acryloyl-l-phenylalanine (PHE) monomer exhibits no significant deviation of the titration curve in the presence and absence of silver ion (Figure 1) in the low pH range involving the ionization of the carboxylic group.

**Figure 2:**
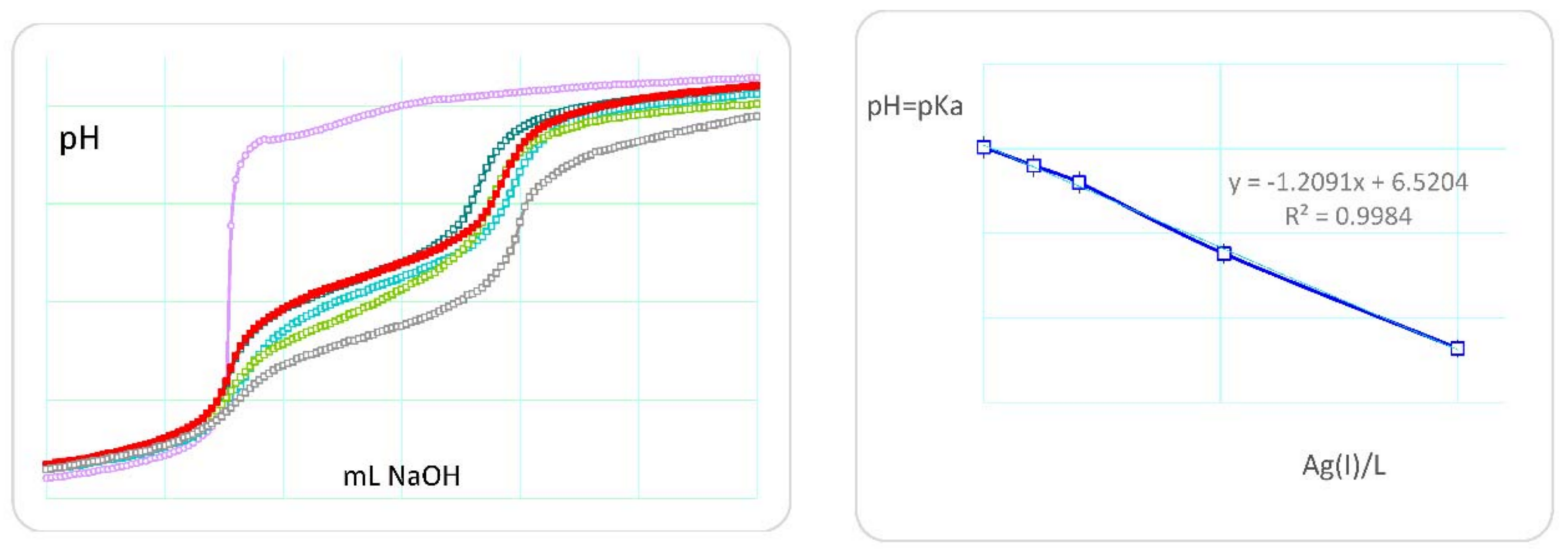
(**Left**) potentiometric titration curves of monomer MHist (L) in the absence (red filled) and in the presence (open circles) of increasing amount of Ag(I) ions. The violet line is only silver(I) ions. (**Right**) plot of the pH = pKa values in relation to the Ag(I)/L molar ratio. L: 0.15 mmol; Ag(I): 0.015, 0.030, 0.075, 0.15 mmol.

The superimposed titration curves (pH vs. mL titrant) show deviations at high pH values attributable to the formation of stoichiometric silver hydroxide. On the other hand, the monomer *N*-methacryloyl-l-histidine (MHist) shows titration curves that vary significantly with a regular increase in the amount of silver ion (Figure 2, left).

In fact, any increase in the Ag(I)/L molar ratio (L is the monomer ligand) is reflected in a decrease in the pH range of the buffer zone involving the ionization of the imidazole nitrogen. An approximate graph of pH = pK_a_ in relation to the Ag(I)/L molar ratio shows a good linearity (Figure 2, right).

**Figure 3:**
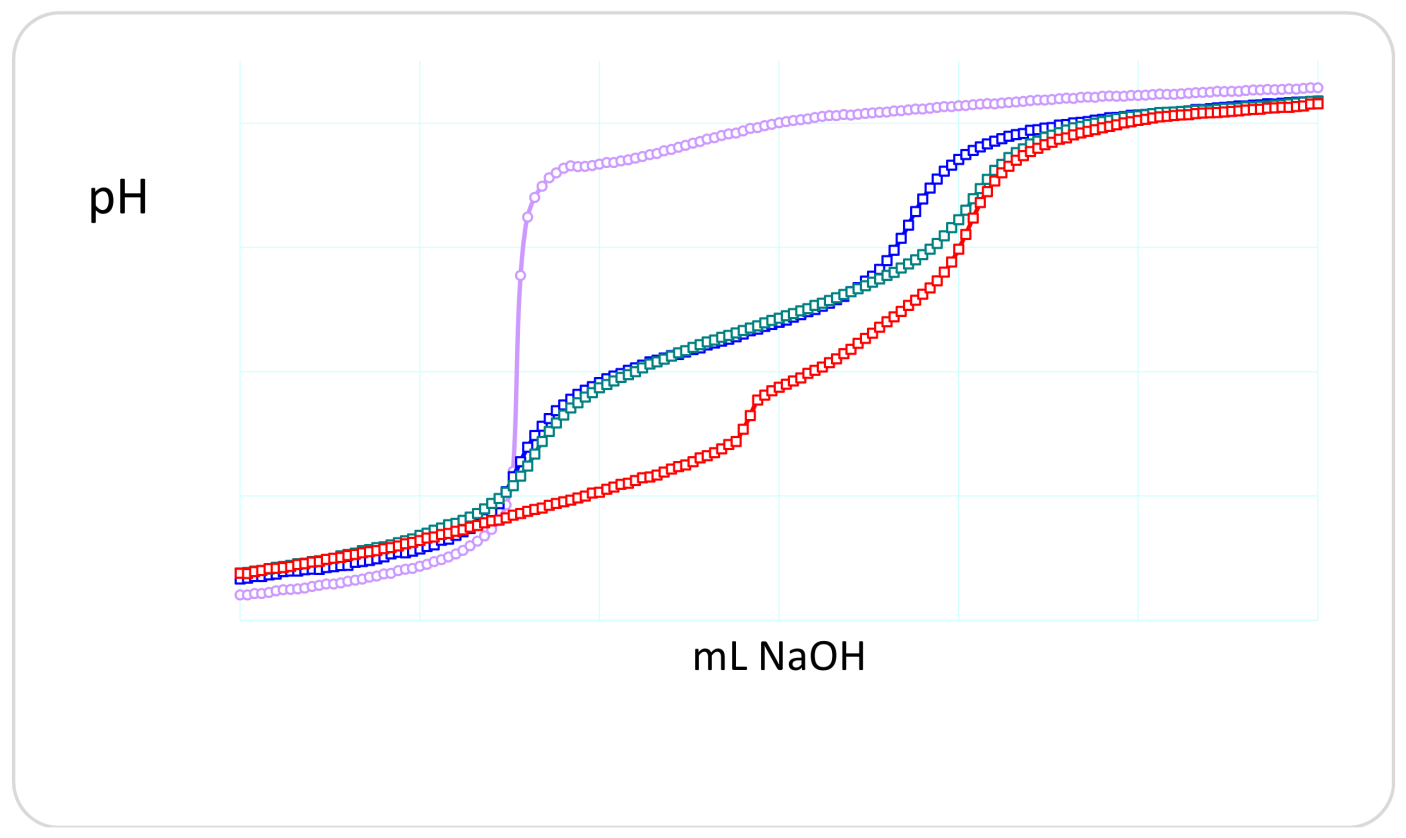
Potentiometric titration curves of poly(MHist-*co*-Nip) without (green) and with (red) silver(I) ions in the Ag(I)/L = 0.5 molar ratio. The titration curve of monomer MHist (blue) and silver(I) (violet) is also reported for comparison.

On the other hand, a copolymer compound, the poly(MHist-*co*-Nip), shows a complexing ability towards the Ag^+^ ions considerably higher than the ligand monomer. As shown in Figure 3, the copolymer protonation behaves exactly like the precursor monomer, whereas in the presence of silver ions a considerable flattening of the titration curve occurs at low pH values and in the pH range that involves the complexing equilibria.

The presence of Ag^+^ ions produces a lowering of the pH as a consequence of its complexation to the ligand; this causes a release of free H^+^ ions in solution from the ligand. The protons released are stoichiometric with respect to the Ag^+^ ions present, which suggests a possible stoichiometry whereby an Ag^+^ ion is complexed to a ligand and two ligands are involved in two complex species with a single Ag^+^ ion. The complexation behavior with the silver ion of the copolymer seems to be similar to that reported in the literature for simple imidazole and histidine, but different to that reported above for the MHist monomer [31,32]. The titration curves of the latter highlight the formation of complexes in the presence of silver ions but are not as discriminated and synergistic as in the case of copolymers. It is reasonable to hypothesize a synergy also for hydrogels and that, therefore, the presence of silver ions favors the interaction of several ligand units towards the metal ion [33]. This is what actually happens and is confirmed by the behavior of the swelling of hydrogels in the presence of the metal ion, as described below.

### 2.2. Syntheses and Characterization

Among the different methods of formation of silver nanoparticles in aqueous solution, we used the reduction of Ag^+^ ion, complexed/absorbed in suitable polyelectrolyte hydrogels, by means of the reducing sodium borohydride NaBH_4_ [29,34]. Usually, this reaction is carried out in the presence of colloidal stabilizers to avoid the aggregation of the nanoparticles. This is a conventional method for the synthesis of nanoparticles that makes use of materials with potential hazards, especially for biomedical applications. There is therefore a general interest in developing environmentally friendly processes to obtain metal nanoparticles through the use of appropriate polyelectrolytes. Recently some polysaccharide hydrogels [35] and some based on chitosan modification [36] have shown reducing properties towards metal ions and in particular Ag^+^ ions; at the same time, they have been shown to stabilize the nanoparticles in colloidal solution. However, the reduction reaction must take place at temperatures far above the ambient temperature. In the case of our hydrogels, their thermosensitive properties limit the temperature rise above their LCST (Lower Critical Solution Temperature). The presence of the Nip units in some hydrogels involves an LCST of just over 32 °C. So a reduction of Ag^+^ ion would see the hydrogel always in a collapsed state. Acrylic hydrogels with Nip units make use of NaBH_4_ for Ag^+^ ion reduction [37]. However, a green process is being developed to synthesize metal nanoparticles from our proposed hydrogels.

**Scheme 2:**
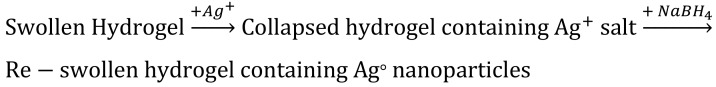
Schematic process of the collapse and swelling of the hydrogel in the presence of silver ions and its subsequent reduction to metallic silver.

In this paper we plan to use the functional groups present on the hydrogel to mitigate the high surface energy of the nanoparticles during the reduction. The functional groups of α-amino acid residues help to stabilize the particles. Furthermore, the silver ion has a higher affinity towards the amine group than the carboxylic group; this involves the formation of relatively stable silver-binding complexes, as suggested by the values of the stability constants [30]. As shown during the synthesis procedure, the interaction between the Ag^+^ ion and the ligand, in the ionized form, leads to a sudden collapse of the swollen hydrogel, as a consequence of a strong electrostatic interaction between opposite charges (Scheme 2).

**Figure 4:**
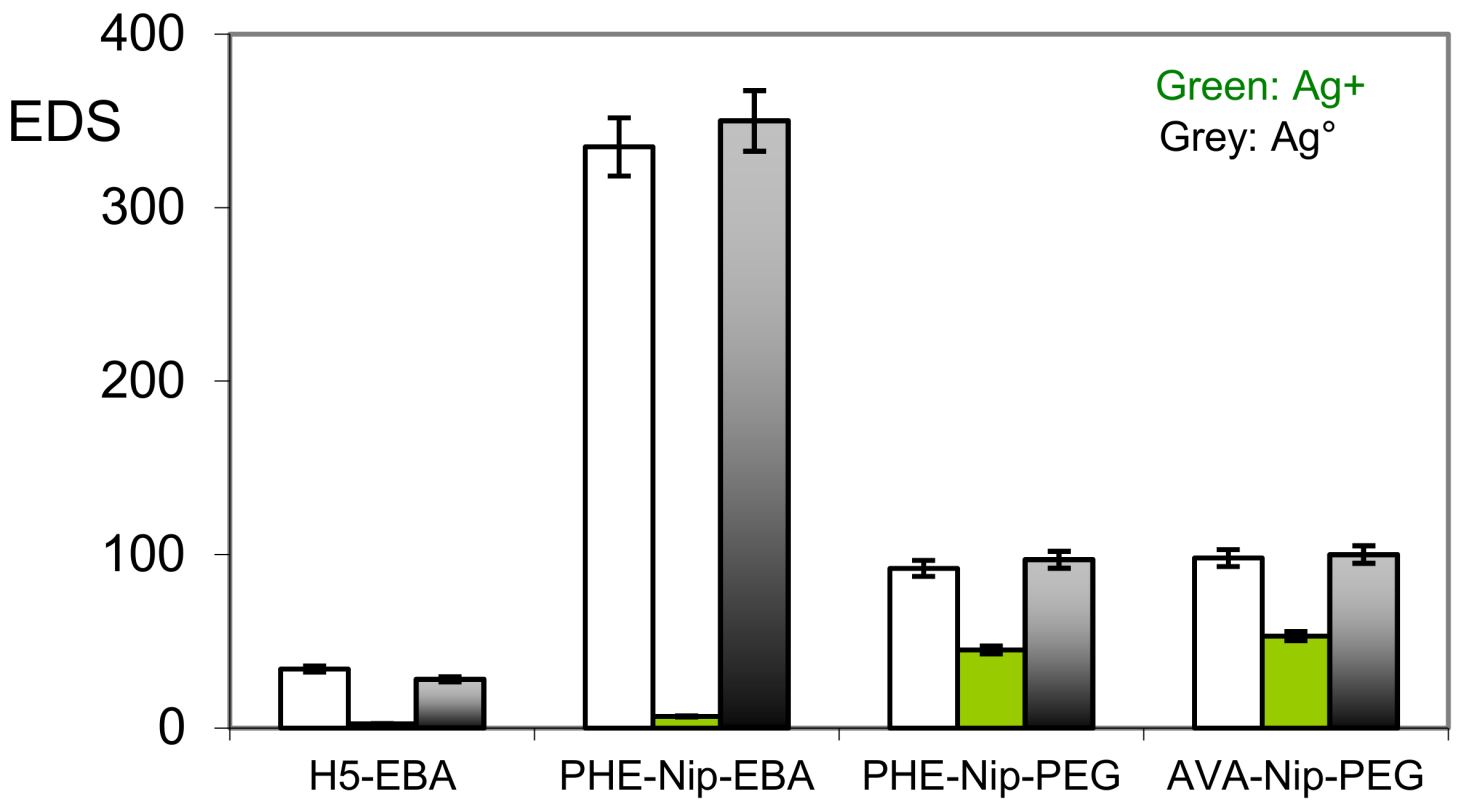
Equilibrium degree of swelling (EDS) of hydrogels in the native state, in the complexation state with silver ion, and in the embedded state with silver nanoparticles.

All four hydrogels considered, despite their different degree of cross-linking, suffer a considerable collapse in the presence of Ag^+^ ions. While the measured value of the equilibrium degree of swelling (EDS) is practically negligible for the zwitterionic hydrogel H5-EBA, the degree of swellability for the other carboxyl hydrogels can be related to the ionic unit content present on the polymer. A lower content of carboxyl groups determines a higher EDS value due to the presence of the *N*-isopropylacrylamide (Nip) non-ion units. This is shown by the comparison of the hydrogels reported in Figure 4.

**Figure 5:**
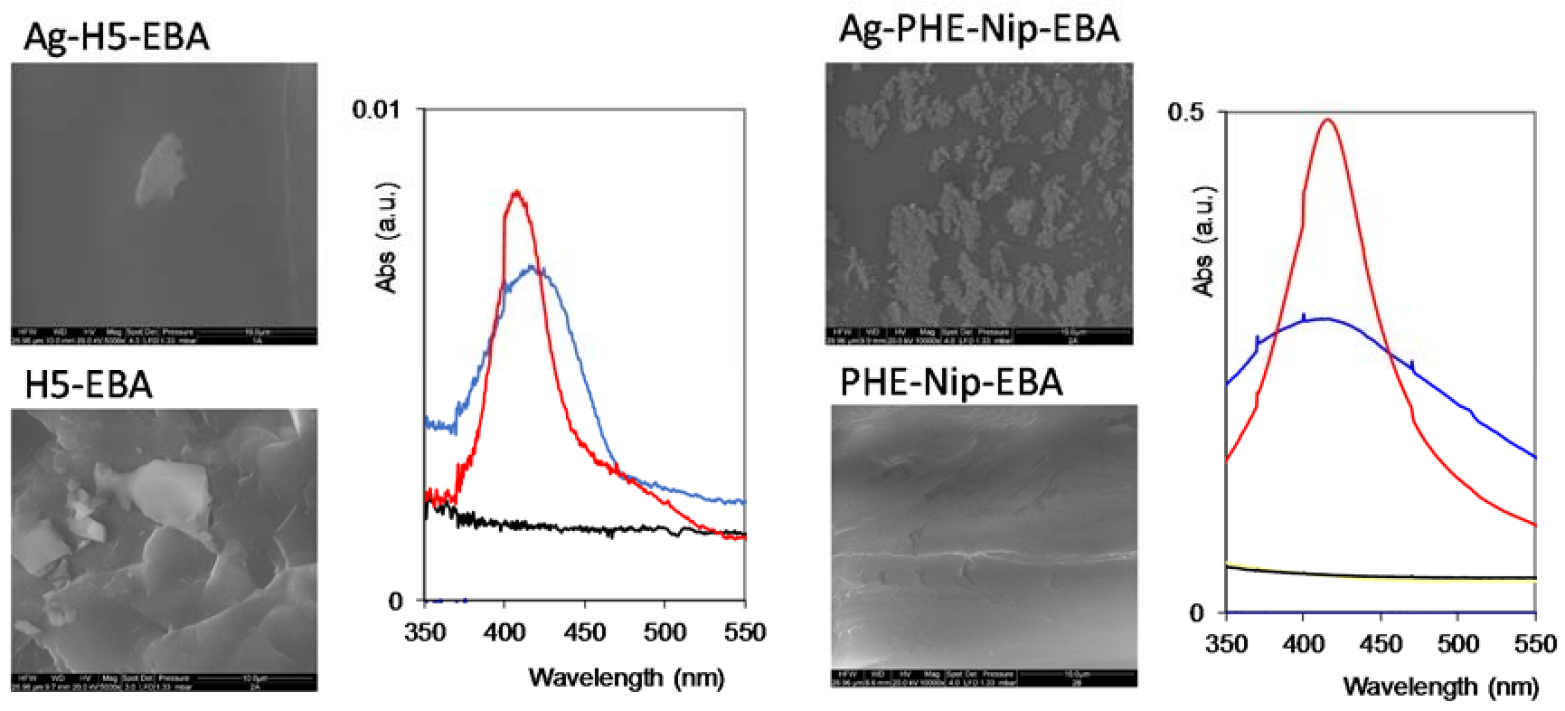
SEM images (scale bar 10 μm) and UV-visible spectroscopy of hydrogels H5-EBA and PHE-Nip-EBA with (above) and without (below) silver nanoparticles. Hydrogels complexed with Ag^+^ (black line), with silver nanoparticles (blue line), and after 24–48 h (red line).

**Figure 6:**
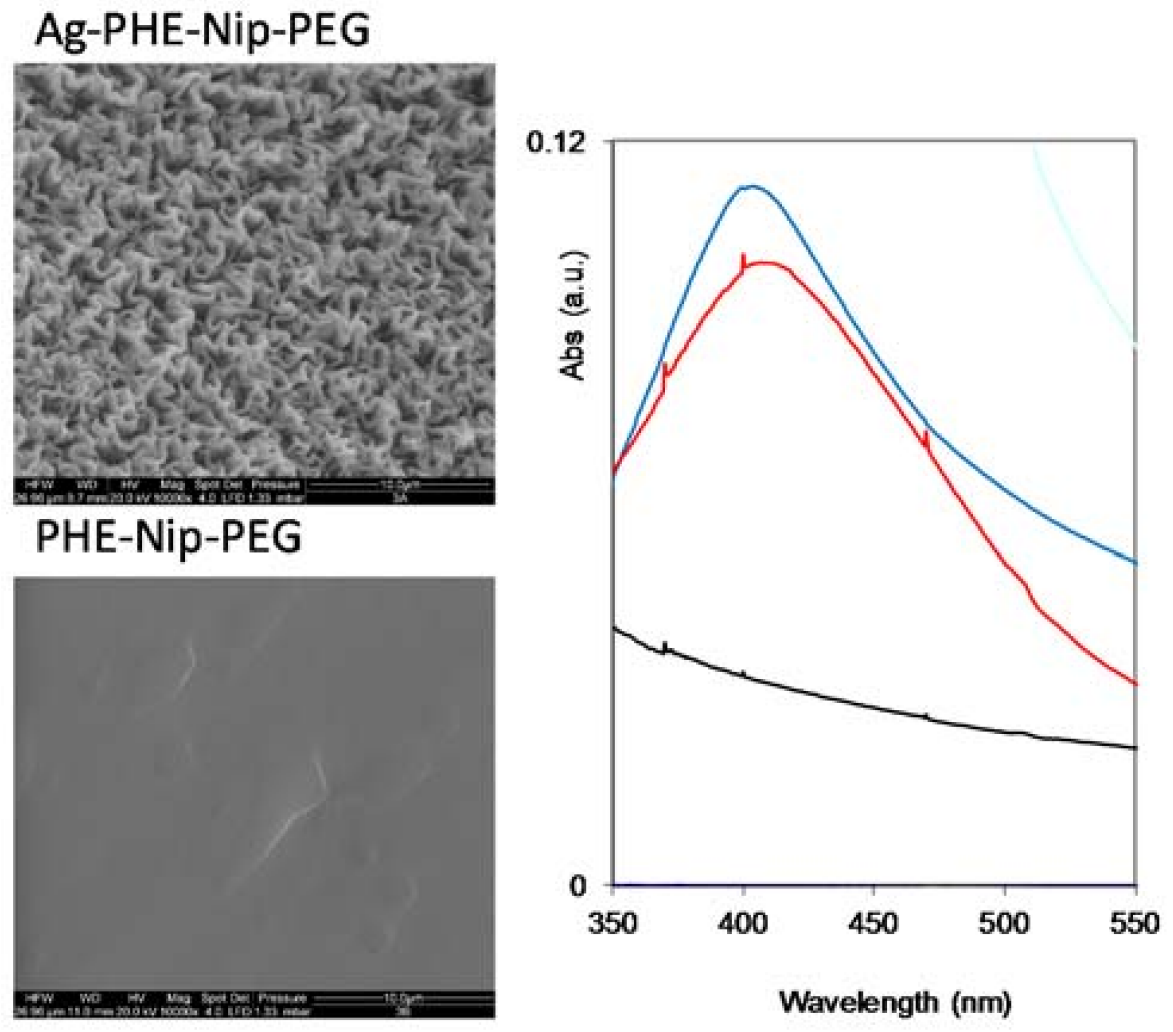
SEM images (scale bar 10 μm) and UV-visible spectroscopy of the hydrogel PHE-Nip-PEG with (above) and without (below) silver nanoparticles. Hydrogel complexed with Ag^+^ (black line), with silver nanoparticles (blue line) and after 24–48 h (red line).

A greater EDS value for PHE-Nip-PEG and AVA-Nip-PEG hydrogels in the presence of Ag^+^ ions is to be attributed to the decreased amount of PHE and AVA ion units, despite their different hydrophobic properties. As a consequence, the collapse of the hydrogel is mainly due to charge neutralization following the Ag^+^ ion complexation with the imidazole nitrogen and the carboxylate ion. On the other hand, the subsequent Ag^+^ ion reduction step with metallic silver, by NaBH_4_, restores the swollen hydrogel state less quickly. This means that the whole Ag^+^ ion is reduced and that the Ag^+^ ion bound to the ligand probably ensures the nucleation of the neighboring silver atoms for the formation of stable silver nanoparticles. The first macroscopic observation of hydrogels, in the presence of metallic Ag, was the significant color difference of the obtained product: black in the case of the H5-EBA hydrogel, while the others are more clearly brown. In any case, even if less evident for Ag-H5-EBA, the external surface of all the dry nanocomposite hydrogels is typically of metallic silver. A topographic evaluation shows the SEM reported in Figure 5 and Figure 6, along with the UV-visible spectra of the hydrogels in the native state, complexed with Ag^+^ ions and silver nanoparticles.

**Figure 7:**
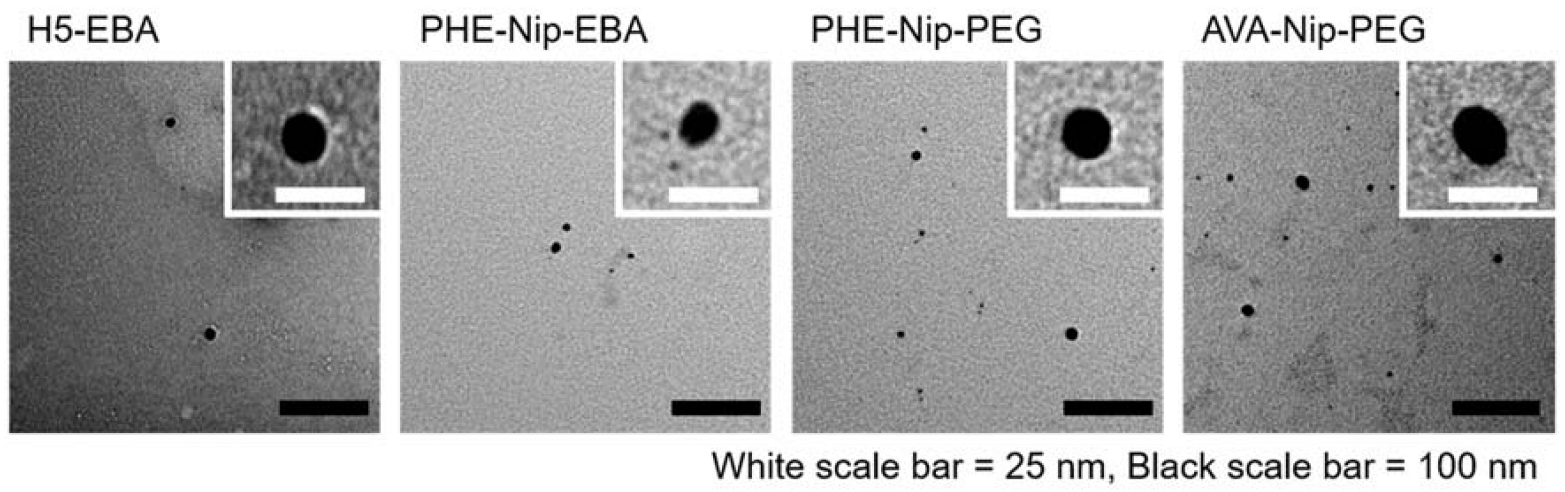
TEM images of the silver nanocomposite hydrogels: H5-EBA, PHE-Nip-EBA, PHE-Nip-PEG, and AVA-Nip-PEG.

In any case, the surface of the nanocomposite hydrogel sample is different from that of the corresponding native hydrogel. While the H5-EBA hydrogel shows greater flattening in the presence of metallic silver, the more homogeneous hydrogel PHE-Nip-EBA shows residual patches probably of externally agglomerated silver. A particular behavior is observed for the PHE-Nip-PEG hydrogel (Figure 6); the homogeneity of the native hydrogel is transformed into crystal-like in the presence of Ag. This point, which may raise a particular interest, requires further investigation. Furthermore, due to the reduction of Ag^+^ ion, the white hydrogel immediately became dark on the surface. To confirm the formation of silver nanoparticles, a UV-visible analysis clearly showed an absorption peak around 400 nm, due to the surface plasmon resonance effect (Figure 5 and Figure 6); this peak is not present in the native and in the Ag(I)-complexed hydrogel [38]. A great deal of information about the physical state of the nanoparticles can be obtained by analyzing the spectral properties of silver nanoparticles incorporate into the hydrogel. The initial UV-visible broad spectrum contracted after 24 h and remained unchanged over time. This time interval allowed us to monitor the formation of the nanoparticles inside the hydrogel that did not undergo any aggregation so that their size remained unaltered. This optical response shows how active hydrogel groups exert a kind of stabilization towards the formed nanoparticles. The spectral response is compatible with a fairly low nanoparticle size. In fact, a TEM analysis (Figure 7) shows that the nanoparticles have a spherical shape of about 15 nm in size and are well distributed within the hydrogel.

It is worth mentioning that in the case of the zwitterionic hydrogel (H5-EBA) the silver nanoparticles are markedly spherical and well-defined, uniformly distributed, and considerably spaced from each other throughout the polymer network. On the other hand, albeit to a lesser extent, carboxyl hydrogels show some irregularities, with sporadic aggregations of different shapes.

**Figure 8:**
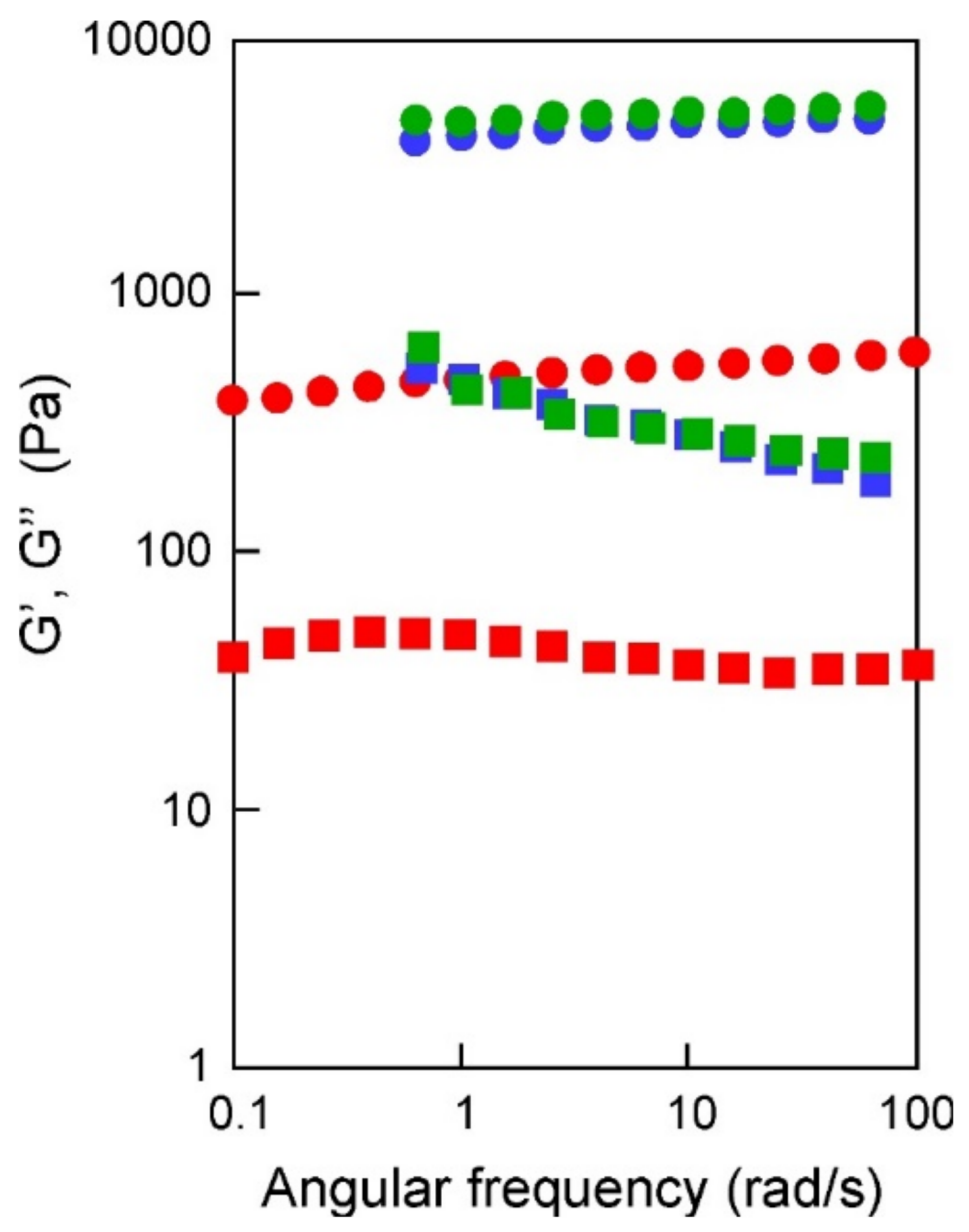
Angular frequency dependence (rad/s) of storage modulus G’ (circles) and loss modulus G” (squares) of silver nanocomposite hydrogels PHE-Nip-EBA (red), PHE-Nip-PEG (blue), and AVA-Nip-PEG (green). Strain 0.1%, 25 °C.

Some dynamic mechanical analysis of nanocomposite hydrogels provided quantitative information on the viscoelastic and rheological properties. Storage elastic modulus G’ and loss elastic modulus G” of the hydrogels were measured, and the viscoelastic characteristics of the anionic hydrogels are reported in Figure 8.

As shown in the figure, all the composites are characterized by a well-developed network, since the storage modulus G’, which exhibits a plateau in the 0.1–100 rad/s frequency range, is always greater than the loss modulus G”. In contrast to the high elastic modulus of PHE-Nip-PEG and AVA-Nip-PEG, the hydrogel PHE-Nip-EBA showed a low modulus. The weaker behavior of the hydrogel PHE-Nip-EBA may be due to the lower crosslinking density. It is known that G’ correlates with the rigidity of hydrogels, so the cross-linked hydrogels PHE-Nip-PEG and AVA-Nip-PEG are strong gels [39]. Moreover, for all the nanocomposites the loss factor (tanδ) was 0.1, which was almost the same as that of a general chemically cross-linked gel. Since the strain characteristics and the angular frequency characteristics showed the same tendency, it was found that similar viscoelastic properties are exhibited except for sample hardness.

### 2.3. In Vitro Antimicrobial Activity

**Figure 9:**
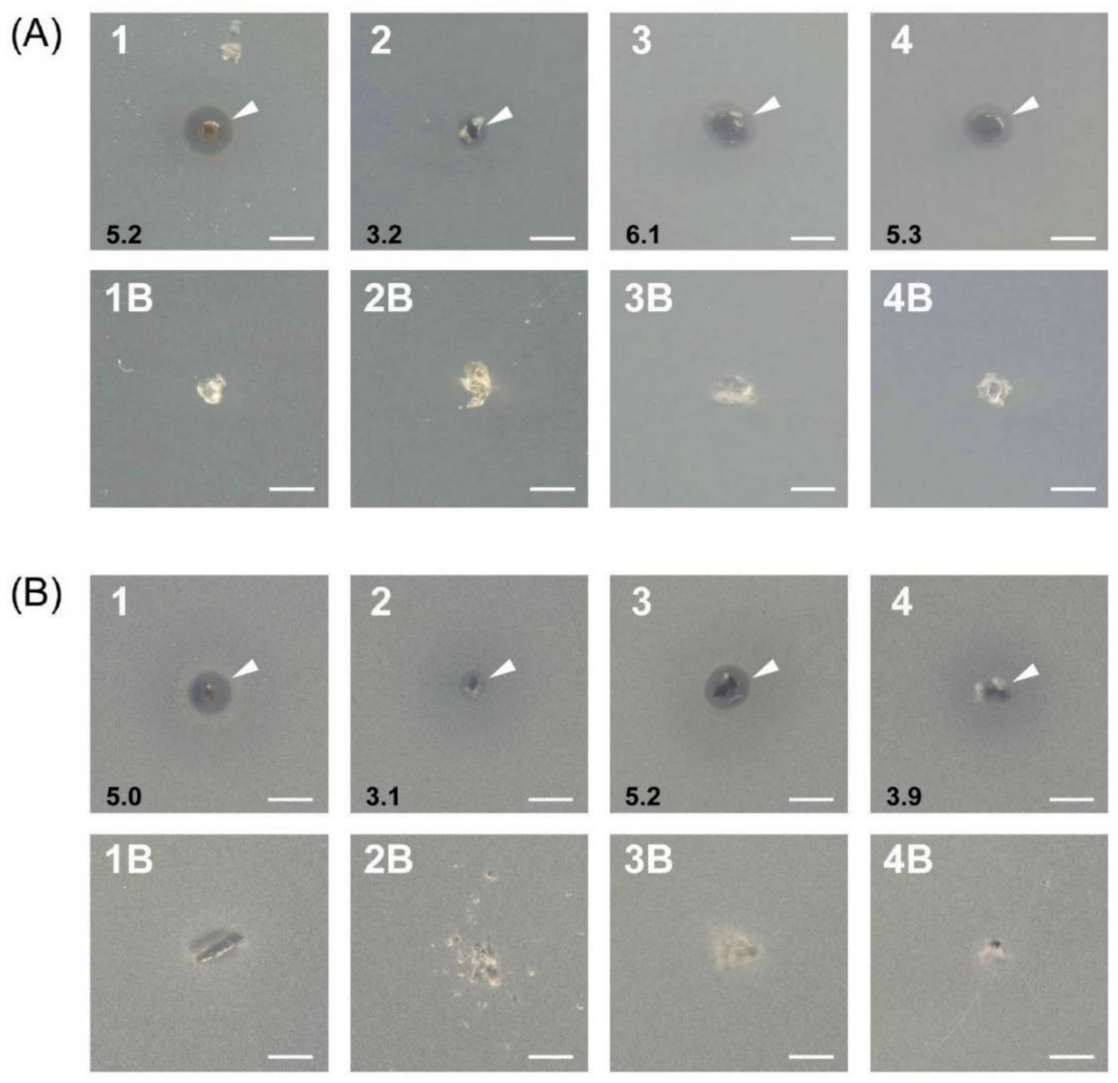

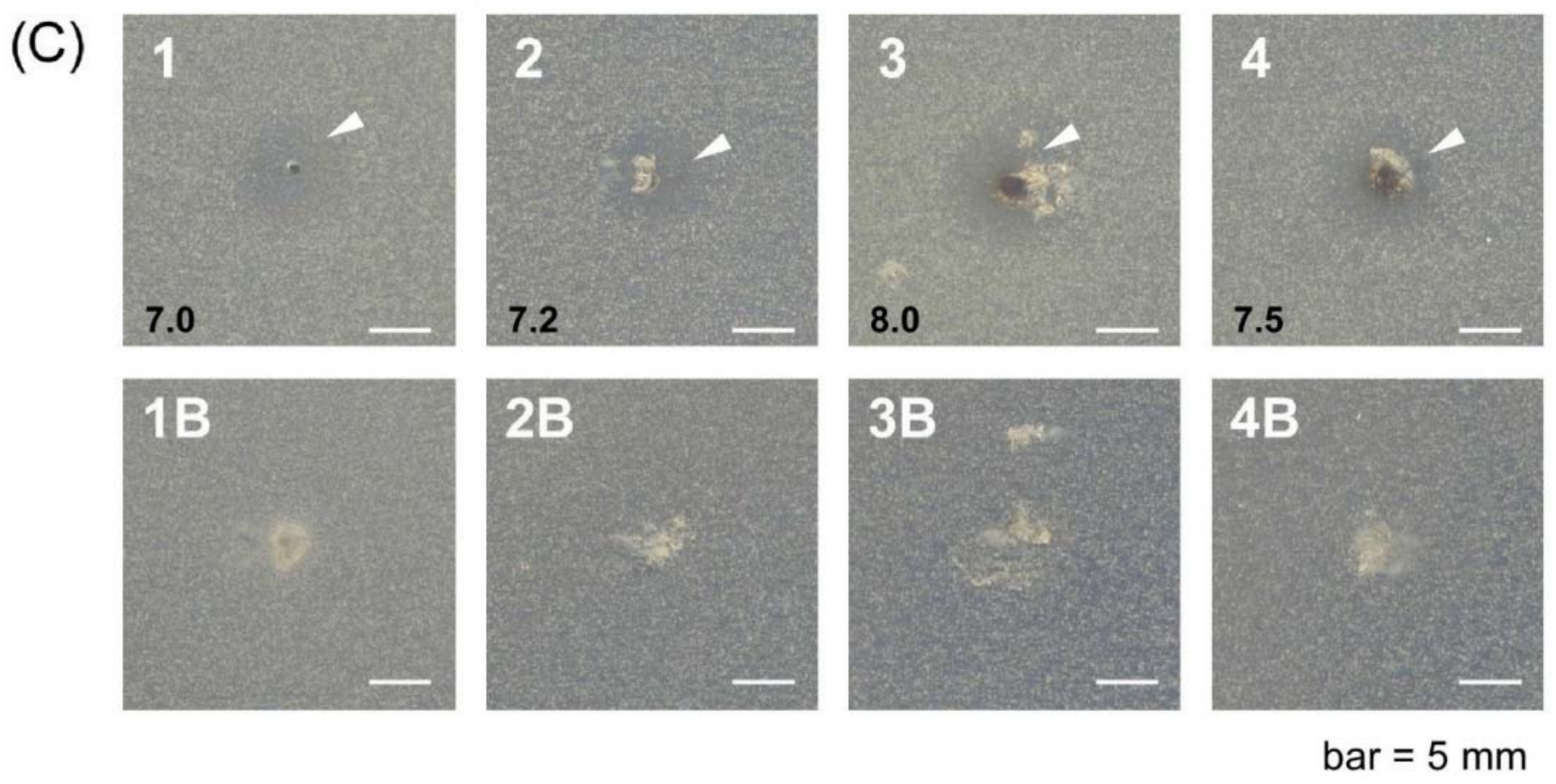
Antimicrobial activities of silver nanocomposite hydrogel (1–4) and native hydrogel (1B–4B) against *Escherichia coli* (**A**), *Bacillus subtilis* (**B**), and *Saccharomyces cerevisiae* (**C**). (Hydrogels: 1, H5-EBA; 2, PHE-Nip-EBA; 3, PHE-Nip-PEG; 4, AVA-Nip-PEG). Arrow heads indicate inhibitory zones, and black numbers mean diameter of inhibitory zone (mm).

The in vitro antibacterial screening of native hydrogels and their silver analogues was carried out against Gram-positive (*B. subtilis*) and Gram-negative (*E. coli*) bacteria, and a fungus (*S. cerevisiae*). The results reported in Figure 9 suggest that the silver nanocomposite hydrogel (1–4, indicated with arrow heads), compared to the corresponding native one (1B–4B), shows inhibitory effects, since a clear inhibition zone was observed under similar conditions. The possible mechanism for this inhibition zone may be only attributed to the silver ion released by the nanocomposite hydrogel containing the silver nanoparticles; the latter could be toxic because they release silver ions, which are well known for their antibacterial and other destructive behaviors [40]. The tests for the release of silver nanoparticles by hydrogels, both in pure water and in phosphate buffer at pH 7.4, have shown that only the PHE-Nip-EBA nanocomposite hydrogel is able to release free silver nanoparticles in solution over time, as evidenced by the increasing spectrophotometric peak at about 400 nm and due to the surface plasmonic effect. Unlike other hydrogels, the low cross-linking content of the PHE-Nip-EBA hydrogel allows the release of nanoparticles, despite their similar size. However, in both cases, hydrogels with silver nanoparticles could interact with the lipid layer of the cell membrane. From the results, we revealed that the silver nanocomposite hydrogel showed activity towards both Gram-positive *B. subtilis* and Gram-negative *E. coli* (Figure 9, A1 and B1, or Figure 9, A3 and B3). Compounds including antibiotics are generally hard to permeate through periplasm, but no significant difference between Gram-positive and Gram-negative bacteria in this study.

Moreover, it was noticeable that the antifungal activity of the silver nanocomposite hydrogels was significantly enhanced. The diameter of the inhibition zone against *S. cerevisiae* was larger than the antibacterial one (Figure 9C (1–4)). This can be a good result for the treatment of contact lenses [25]. Fungal keratitis is a severe ocular disease that could cause blindness and is very often observed in developing countries. It is known that the variety of the forms and characteristics of various silver nanoparticles are also responsible for differences in their antibacterial mode of action and probably bacterial mechanism of resistance [17]. The size and shape of the silver nanoparticles from the present hydrogels might be useful for their antifungal effects.

## 3. Conclusions

Silver nanoparticles are versatile in different technologies and can be incorporated into many materials because of their functionality, especially optical and antibacterial. Hydrogels, which have attracted great interest in the field of controlled drug delivery, may be an appropriate material for incorporating silver nanoparticles with useful biomedical devices for the continuous release of a low level of Ag^+^. The formation of silver nanoparticles was carried out on some polyelectrolyte hydrogels bearing α-amino acid residues, without the use of stabilizing agents. The zwitterionic hydrogel, containing residues of l-histidine, showed greater complexing ability with Ag^+^ ions compared to hydrogels with carboxyl groups. Thus, the imidazole group, as well as having other polymer functionality, can mitigate the stabilization of Ag nanoparticles, thereby decreasing the surface energy. These hydrogels, already experimented on for the release of drugs, needed a further control to provide protection against bacteria. The Ag^+^ salt reduction reaction with NaBH_4_ made it possible to verify that silver nanoparticles of optimal size, shape, and distribution can be easily prepared for useful applications, since their antibacterial and antifungal properties have been verified. In fact, the latter were very powerful as gram+/gram− bactericides, and much more effective in their antifungal activity against *Saccharomyces cerevisiae*.

## 4. Materials and Methods

### 4.1. Materials

Monomers (Hist, PHE, and AVA) used for the synthesis of hydrogels were obtained in a similar way to that described in previous papers [41,42,43]. The synthesis of the monomer MHist and the poly(MHist-*co*-Nip) was previously reported [44]. Cross-linking agents (EBA and PEG-DA), and *N*-isopropylacrylamide (Nip) were purchased from Sigma-Aldrich (St. Louis, MO, USA). While the silver nitrate salts was from Carlo Erba (99.9% purity, Milan, Italy), the sodium borohydride (99% purity), as well as all the other salts and reagents used for the buffer solutions and for the synthesis of the hydrogels, were from Sigma-Aldrich.

### 4.2. Synthesis of Hydrogels

The hydrogels reported in the present work have been obtained by radical polymerization of the corresponding synthetic and commercial monomers, using EBA and PEG-DA (Mn 258) as cross-linking agent. Their synthesis has been reported in previous papers and Table 1 summarizes the monomeric composition [6,11,12]. Apart from the zwitterionic and homopolymer hydrogel H5-EBA, the other three hydrogels (PHE-Nip-EBA, PHE-Nip-PEG, and AVA-Nip-PEG) are copolymers of *N*-isopropylacrylamide and vinyl monomers bearing the carboxyl functional group. The latter were crosslinked with EBA and with PEG-DA in different molar ratios.

Samples of hydrogels thus obtained have been used to produce the formation of silver nanoparticles distributed within their network. For this purpose we used a chemical reduction method that involved the reduction of silver from its salt (AgNO_3_) in aqueous solution and by an effective reducing agent. A sample of native hydrogel was made to swell in water up to its equilibrium degree of swelling. Subsequently, the hydrogel, separated from the excess water, was added to a silver nitrate solution containing an excess of silver ions with respect to the stoichiometric amount required by the hydrogel. The solution was placed in the dark to react for 24 h. Table 2 shows the experimental conditions for the preparation of the nanoparticles, without the aid of stabilizing agents. After 24 h, the residual of collapsed hydrogel was filtered on a Strainer cell (70 μm pore size) and repeatedly washed with twice-distilled water. The hydrogel, once dried but in the moist state, was placed in contact with an aqueous solution of NaBH_4_ of suitable concentration (Table 2). The variation to an intense dark color of the colorless hydrogel showed the immediate formation of reduced silver throughout the hydrogel. Unlike the intense brown color shown by the three anionic hydrogels, the H5-EBA hydrogel immediately revealed an intense black color. The hydrogels were allowed to react in the dark for 24 h, observing a constant swelling over time. Then, after filtering on a Strainer cell, the swollen hydrogels were repeatedly washed with twice-distilled water until the characteristic UV-visible absorption around 400 nm, due to the surface plasmonic effect, disappeared [45]. The hydrogels were dried and placed to dry first in the air and then under a vacuum. A significant increase in weight was observed only for the two hydrogels cross-linked with PEG-DA, i.e., PHE-Nip-PEG and AVA-Nip-PEG.

### 4.3. Swelling

The swelling of the hydrogels, from the native state to the complexed state with silver ions and finally with silver nanoparticles, was in three steps and involved the reduction of the silver ion with sodium borohydride to obtain silver nanoparticles incorporated into the polymer network [29,34]. First, a weighed sample of dry hydrogel, placed in a Strainer cell (70 μm pore size) at room temperature, was made to swell in slightly basic water with a diluted sodium hydroxide solution. After the complete swelling, the hydrogel was thoroughly washed with distilled water for a few days and blotted with tissue paper to remove any surface water. The equilibrium degree of swelling (EDS) was calculated from the weight of the wet sample (W_wet_) using the following equation:

EDS = (W_wet_ − W_dry_)/W_dry_,

where W_dry_ is the weight of the dry hydrogel sample before the swelling. Second, the hydrogel was immersed in a suitable aqueous silver nitrate solution (a hydrogel collapse was immediately observed) and left for 24 h. After this time, the hydrogel was washed with twice-distilled water, blotted with tissue paper, and weighed. In the third step, the collapsed hydrogel was placed in a suitably prepared fresh aqueous solution of sodium borohydride. The prompt reaction led the hydrogel to become dark and floating in a yellow solution. The reaction was run in the dark for 24 h (slow and progressive swelling was noted). Subsequently the hydrogel, with the silver nanoparticles, was repeatedly washed and left in twice-distilled water overnight. After further washing, the hydrogel was blotted with tissue paper and weighed. The value of EDS was thus calculated by the corresponding W_wet_, by the above equation.

### 4.4. Potentiometric Titrations

Potentiometric titration curves were obtained with a TitraLab 90 automatic titrator (Radiometer Analytical, Lyon, France), similar to that previously reported [43]. The titrator, equipped with the TIM900 (a powerful Titration Manager) and a high-precision autoburette, was controlled by TimTalk 9 (Windows-based software). Measurements were carried out in 0.15 M NaClO_4_ solution (50 mL) in a glass cell thermostated at 25 °C and controlled by a temperature sensor (T201). The aqueous solution, containing a weighed quantity of dissolved ligand together with silver nitrate and a known volume of standard HNO_3_ solution, was titrated with a standard NaOH solution (0.0554 mol/L) under a nitrogen stream to avoid CO_2_ contamination. A combined pH electrode (Red Rod, Loveland, CO, USA), calibrated with two buffers (pH 4.01 and 7.00, from Radiometer, Copenhagen, Denmark) before each measurement, controlled the titrations with a stepwise addition of titrant (0.04 mL) every 240 s.

### 4.5. Spectroscopic Characterization

The main spectroscopic characterization of the hydrogels (with and without silver nanoparticles) was done by transmission electron microscopy (TEM), scanning electron microscopy (SEM), and UV-visible spectroscopy. TEM images were taken using a JEOL JEM-1230 at an accelerating voltage of 80 kV with supporting by the Materials Characterization Support Unit, RIKEN CEMS, Hirosawa, Japan. Each gel powder was swollen by milliQ water (Millipore, Billerica, MA, USA) and then sonicated. A drop (3 μL) of dispersion was mounted on a carbon-coated Cu grid (Okenshoji Co., Ltd., Tokyo, Japan) and stained negatively with 2% samarium acetate, followed by the suction of the excess fluid with a filter paper. SEM images examined (at 20 kV and at different magnifications) the morphology of hydrogels before and after the formation of silver nanoparticles. It was performed by XL20 Philips (North Billerica, MA, USA). scanning electron microscopy, where the sample was mounted on SEM stubs with Leit-C Conductive Carbon Cement (West Chester, PA, USA) and sputtered with 20 nm gold by a Balzers Med 01 sputter-coater. The UV-visible spectra of the swollen hydrogels (native, with silver ions, and silver nanoparticles) were recorded with a Specord 210 spectrophotometer (Analytikjena, Germany) equipped with 10 mm quartz cuvettes. A sample of hydrogel, well washed with distilled water and then dried, was introduced into the quartz cuvette and the UV-visible spectrum was recorded in the 350–800 nm range. Samples containing silver nanoparticles showed an absorption band around 400 nm, characteristic of the plasmonic surface effect.

### 4.6. Rheological Measurements

Viscoelastic properties of the nanocomposite hydrogels were measured using the AR-G2 rheometer (TA instruments, New Castle, DE, USA). The hydrogel was swollen in MilliQ water for 24 h. The strain dependency was measured at an angular frequency of 1 rad/s. The angular frequency dependency was measured at a swing angle of 0.1%.

### 4.7. Antibacterial and Antifungal Activity Studies

Antibacterial activity screen studies of native and their corresponding silver nanocomposite hydrogels were carried out by a direct method. Agar media (LB for *Escherichia coli* (Gram−) and *Bacillus subtilis* (Gram+), YPD for *Saccharomyces cerevisiae* (Yeast)) were mixed with a preculture of test microorganisms to obtain the optical density at 0.1, and spread to ϕ100-mm plastic dishes. Samples of hydrogels with/without silver nanoparticles were put onto agar media, and the plates were incubated at 37 °C for *E. coli* or 30 °C for *B. subtilis* and *S. cerevisiae* for 24 h. The inhibitory zones that appeared around the hydrogel sample were observed.

## Figures and Tables

**Table 1:** Composition of monomers in the α-amino acid acid-based hydrogels.

Hydrogel	Monomer Composition (mol %)				Cross-Linking Composition (mol %)	
	Hist	PHE	AVA	Nip	EBA	PEG-DA258
H5-EBA	100	-	-	-	5	-
PHE-Nip-EBA	-	50	-	50	2	-
PHE-Nip-PEG	-	10	-	90	-	12
AVA-Nip-PEG	-	-	10	90	-	12

H5-EBA: homopolymeric hydrogel made of *N*-acryloyl-l-histidine (Hist) and cross-linked with *N*,*N’*-ethylene-bisacrylamide (EBA); PHE-Nip-EBA: copolymeric hydrogel made of *N*-acryloyl-l-phenylalanine (PHE) and *N*-isopropylacrylamide (Nip) and cross-linked with EBA; PHE-Nip-PEG: copolymeric hydrogel made of *N*-acryloyl-l-phenylalanine (PHE) and *N*-isopropylacrylamide (Nip) and cross-linked with poly(ethyleneglycol)diacrylate (PEG-DA258); AVA-Nip-PEG: copolymeric hydrogel made of *N*-acryloyl-l-valine (AVA) and Nip and cross-linked with PEG-DA258.

**Table 2:** Experimental conditions for the preparation of silver nanoparticles embedded hydrogels.

Hydrogel	Dry Sample	EDS ^1^ (Swollen)	Conc. Ag^+^ (mM)	Residence Time (h)	EDS ^2^	Conc. NaBH_4_ (mM)	EDS ^3^	Ag° Increase (wt %)
H5-EBA	10.5	34.3	13.5	24	2.5	40.9	27.7	-
PHE-Nip-EBA	9.2	335.0	6.5	24	6.7	38.3	350.0	-
PHE-Nip-PEG	126.3	91.4	10.6	24	45.2	34.0	97.9	4.2
AVA-Nip-PEG	98.1	98.5	10.6	24	53.4	34.0	100.3	7.4

Dry sample: weighed amount (mg) of native dry hydrogel; EDS ^1^ (equilibrium degree of swelling) = (W_wet_ − W_dry_)/W_dry_ of the native sample (where W_wet_ and W_dry_ is the weight of the wet and dry hydrogel, respectively). Conc. Ag^+^: initial concentration of a silver nitrate aqueous solution added to the swollen hydrogel; Residence time: the time left in contact with the hydrogel; EDS ^2^: the measured degree of swelling of collapsed hydrogel in contact with Ag^+^. Conc. NaBH_4_: initial concentration of sodium borohydrate in contact with the collapsed hydrogel; EDS ^3^: the measured degree of swelling of silver nanoparticles embedded hydrogels; Ag° increase: weight increase (%) due to the presence of silver nanoparticles.
